# The Relationship Between Gestational Diabetes and the Risk of Cancer: A Systematic Review

**DOI:** 10.7759/cureus.53328

**Published:** 2024-01-31

**Authors:** Ethan Slouha, Kaitlyn M Gates, Hanin Al-Geizi, Esther Baah, Lucy A Clunes, Theofanis F Kollias

**Affiliations:** 1 Anatomical Sciences, St. George's University School of Medicine, St. George's, GRD; 2 Pharmacology, St. George's University School of Medicine, St. George's, GRD; 3 Microbiology, Immunology, and Pharmacology, St. George's University School of Medicine, St. George's, GRD

**Keywords:** breast cancer, cancer risk, gestational diabetes mellitus, leukemia, thyroid cancer

## Abstract

Gestational diabetes mellitus (GDM) is one of the most common endocrine disorders to occur during pregnancy due to the increase in circulating human placental lactogen (hPL) and possible beta-cell sensitivity. While GDM can be managed either with diet and exercise or pharmacological interventions, it is associated with significant maternal and neonatal complications. Maternal complications include short- and long-term conditions such as pre-eclampsia, preterm birth, arrest of labor, future development of type 2 diabetes mellitus (T2DM), and cardiovascular disorders. Neonates can develop hypoglycemia and hypocalcemia and have a large gestational age (LGA). New research has also highlighted another possible long-term complication for both mothers and offspring, which is the development of cancer. Cancer has various types of progression, but most cause systemic symptoms leading to a reduced quality of life. Cancer can be terminal and can affect the majority of the population; thus, significant effort is being employed to try and reduce its occurrence. This systematic review was conducted with adherence to the Preferred Reporting Items for Systematic Reviews and Meta-Analyses (PRISMA) guidelines using PubMed, ScienceDirect, and ProQuest databases. Initially, 136,019 publications were identified. Through the screening process, a total of 27 publications were finalized within the scope of this paper.

Most studies observing maternal cancer with a history of GDM found that there was an association between the increased risk of cancer and GDM. Specifically, these studies identified the association of GDM with breast, ovarian, cervical, and uterine cancer, as well as other non-reproductive organs such as the thyroid and pancreas. Cancer development in the offspring also presented an association with mothers who developed GDM. The most prevalent cancer evaluated was leukemia, and it was specifically associated with a maternal history of GDM. With the consistent rise in the incidence of cancer, any attempts to reduce its development are imperative to assess. While GDM is essentially a temporary condition that resolves following pregnancy in most patients, the possibility of contributing to future conditions years after its occurrence creates a sense of urgency and necessity to reduce the incidence of GDM. Researchers should be able to identify other unknown biomarkers that contribute to the development of cancer in mothers who experienced GDM as well as their infants.

## Introduction and background

Gestational diabetes mellitus (GDM) is hyperglycemia recognized during pregnancy with a 75 mg oral glucose tolerance test reading 10 mmol/L at 1 hour and 8.5 mmol/L at 2 hours [1]. It is assessed, barring previous history, obesity status, or family history, between 24 and 28 weeks of gestation [1,2]. It is one of the most common complications of pregnancy, affecting up to 15% of pregnancies globally [1,2]. The incidence of GDM increases significantly with age, gestation, obesity, and polycystic ovary syndrome (PCOS) [1, 2]. Markers such as afamine, 1,5-anhydroglucitol, and adiponectin have been identified as potential predictors of the development of GDM [1]. Gestational diabetes mellitus develops due to increased insulin resistance during pregnancy due to increased human placental lactogen (hPL), leading to fatty acid breakdown [1,2]. Beta-cell dysfunction has also been suspected due to hypertrophy and proliferation during the first two trimesters of pregnancy, but this possible mechanism proves insufficient as it does not apply to all aspects of GDM [1,2]. The first line of treatment for GDM is modifying the patient's diet and encouraging light activity following meals [1]. Due to the need for weight gain during pregnancy, weight loss is contraindicated for management [1]. Insulin is used primarily if pharmacological intervention is required, as it does not cross the placenta [1]. Metformin, an oral antidiabetic drug, can also be administered and has been shown to improve a patient's GDM status [1].

The consequences of GDM on the mother include both short- and long-term health issues such as preterm birth, pre-eclampsia, the development of type 2 diabetes mellitus (T2DM) postpartum, and a predisposition to cardiovascular disease [2]. Neonatal complications that occur in mothers with GDM consist of large gestational age (LGA), hypoglycemia, hypocalcemia, macrosomia leading to shoulder dystocia, and hyperbilirubinemia [2]. Recently, there have been studies evaluating long-term complications of GDM in mothers and offspring; one such complication is the development of cancer. A couple of cancers that have been evaluated in the mother are breast cancer and gynecological cancers, with breast cancer being the most common cancer diagnosed in females [3]. While the majority of cervical cancer results from human papillomavirus (HPV), there are cases of cervical cancer that stem from other origins, and overall, it is widely known as a preventable cancer [4]. There has also been an association with the offspring of mothers with GDM developing leukemia, which is the most common pediatric cancer [5].

Objective

Cancer is one of the leading causes of death in older age groups and affects millions. Cancer also greatly hinders the quality of life of those affected, as it leads to systemic symptoms as well as the side effects of the treatment of cancer. There is a global effort to try and reduce the number of individuals who develop cancer, as well as find ways to be able to identify those at risk. As GDM is a common complication in pregnancy, it is imperative to assess its association with the development of cancer in both the mother and offspring. This paper aims to identify the incidence, risk, and common types of cancer that may develop in the mother and offspring in the setting of a GDM diagnosis. The purpose is to bring about awareness of a possible association and to press the need to evaluate further predictors and risk factors associated with developing GDM to reduce the long-term complications of cancer.

## Review

Methods

This systematic review strictly followed the Preferred Reporting Items for Systematic Reviews and Meta-Analyses (PRISMA) guidelines as presented by Liberati et al. [6]. The search was conducted on the PubMed, ScienceDirect, and ProQuest databases from January 1, 2003, to December 1, 2023. The following keywords were used to tailor the search: ‘gestational diabetes mellitus and cancer’, ‘gestational diabetes mellitus and maternal cancer’, and ‘gestational diabetes mellitus and pediatric cancer’. 

Inclusion and Exclusion Criteria

Studies performed on humans, peer-reviewed observational studies, full-text availability, articles focused on the association of GDM and cancer and those published between 2003 and 2023 were included in the study. Articles published before 2003, written in a language other than English, and duplicate publications were excluded.

**Figure 1:**
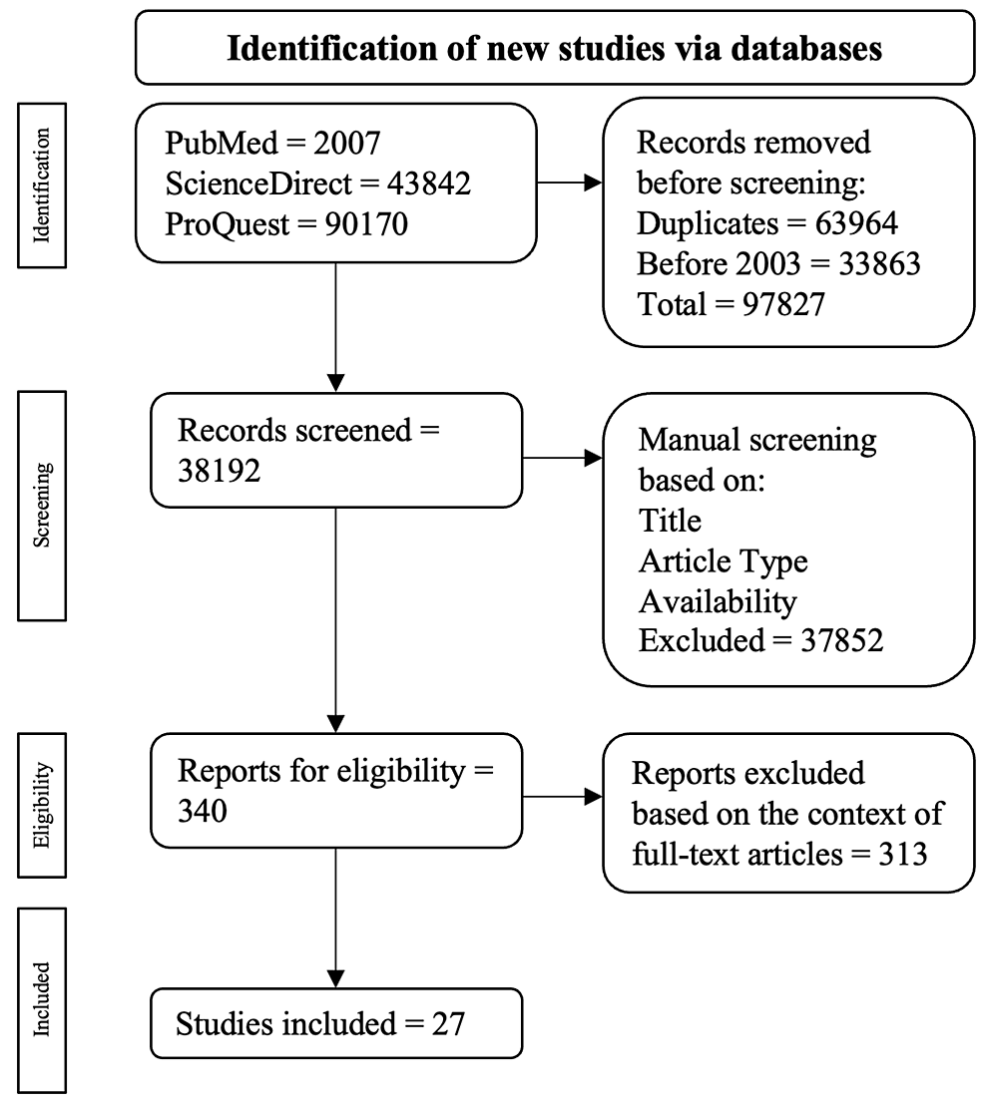
PRISMA flowchart depicting the algorithm employed based on the inclusion and exclusion criteria PRISMA: Preferred Reporting Items for Systematic Reviews and Meta-Analyses

A total of 136,019 publications were initially found (2007 from PubMed, 43,842 from ScienceDirect, and 90170 from ProQuest). Among the exclusions, 63,964 were duplicate publications, and 33,863 were published before 2003. This led to 97,827 being excluded at the end of the automatic screening process, leaving 38,192 publications to be assessed manually. Publications were then manually appraised based on their study type, title, and full-text availability, leaving 340 publications for full-text evaluation. Ultimately, 27 publications were selected. The screening process is displayed in Figure 1.

Bias

The majority of studies presented ample sample sizes and detailed explanations of their methods and protocols. As such, a moderate rating of bias was assigned using the grading of recommendation, development, and evaluation (GRADE) scale. This scale functions to assess imprecision and publication type.

Results

Concerning mothers, the majority of studies observed that there was an association between the development of GDM and the development of cancer as a long-term complication. Common gynecological cancers associated with GDM were breast, cervical, uterine, and ovarian cancers. Also, some studies have observed an association between both thyroid and pancreatic cancer and GDM. In the offspring, GDM was also associated with an increase in leukemia, specifically acute lymphocytic leukemia (ALL). In some cancers, there’s been some evidence of gender differences as well, but not enough research has been done to validate these claims. Table 1 summarizes the articles reviewed in this article.

**Table 1:** Summary of articles assessed in this review GDM: Gestational diabetes mellitus; ER: Estrogen receptor; ALL: Acute lymphoblastic leukemia

#	Author	Country	Design & Study Population	Findings	Conclusion
1	Bertrand et al., 2022 [7]	USA	Retrospective cohort study (n = 257,290)	There was no greater increase in breast cancer in GDM mothers under 55 years old than in those without GDM.	Parous mothers with GDM failed to show an increased risk of young-onset (<55 years) breast cancer than those without GDM. Parity was maintained as a protective factor for mothers with and without GDM.
2	Deleskog et al., 2017 [8]	Sweden	Retrospective cohort study (n = 4,239,965)	Maternal type 1 diabetes and GDM were linked to a reduced risk of congenital brain tumors and an increased risk of leukemia.	An offspring born to mothers with GDM had a decreased risk of developing congenital brain tumors and an elevated risk of leukemia when compared to the offspring whose mothers did not have GDM.
3	Sella et al., 2011 [9]	Israel	Retrospective cohort study (n = 185,315)	The risk of developing pancreatic cancer and hematological malignancies was significantly higher in mothers with GDM.	GDM is linked to a heightened risk of developing pancreatic cancer and hematologic malignancies.
4	Fuchs et al., 2017 [10]	Israel	Retrospective cohort study (n = 104,715)	There was a higher cumulative incidence in patients with a history of GDM, and GDM remained an independent risk factor for female malignancies.	At follow-up, the patients with GDM demonstrated a significantly increased risk of uterine, ovarian, and breast cancer.
5	Han et al., 2018 [11]	Korea	Retrospective cohort study (n = 102,900)	The incidence of cancer was significantly higher in mothers who had GDM, specifically thyroid cancer and breast cancer. Only thyroid cancer was positively associated with GDM.	GDM may only be associated with an increased risk of developing thyroid cancer.
6	Peng et al., 2019 [12]	Taiwan	Prospective cohort study (n = 990,572)	There was a higher incidence of cancer in mothers who had GDM during pregnancy than those who did not.	GDM is a risk factor for the development of different types of cancer.
7	Dawson, 2004 [13]	New Zealand	Cohort study (n = 753)	4.5% were hospitalized with a confirmed diagnosis of malignant neoplasm. 2.39% were cases of malignant neoplasms of the breast. There was a significant association between gestational glucose intolerance and the presence of malignant neoplasms, particularly malignant neoplasms of the breast.	Glucose intolerance during pregnancy was linked to an increasing risk of malignant neoplasms, particularly malignant neoplasms of the breast.
8	Pace et al., 2020 [14]	Canada	Retrospective cohort study (n = 34,294)	After adjustments were made, there was no increased risk of developing cancer overall in mothers with GDM. However, there was a higher risk of thyroid cancer in those who had GDM.	No association was found between a history of GDM and an overall risk of cancer. However, there was a higher risk of thyroid cancer in mothers who developed GDM.
9	Park et al., 2017 [15]	USA	Prospective cohort study (n = 39,198)	Having GDM during one pregnancy was not determined to be a risk factor for developing cancer; however, mothers with two or more pregnancies with GDM were more likely to develop breast cancer, specifically ER+ breast cancer.	Having GDM in two or more pregnancies was significantly associated with developing breast cancer, specifically ER+ breast cancer. There is a suggestive relationship between GDM in one pregnancy and developing ER- breast cancer.
10	Bejaimal et al.2015 [16]	Canada	Retrospective cohort study (n = 149,049)	There was no significant increase in the risk of cancer between mothers with GDM and cancer, except for thyroid cancer.	Mothers with GDM did not have a higher cancer risk overall during their first 10 years postpartum.
11	Bertrand et al., 2020 [17]	USA	Prospective cohort study (n = 41,767)	No association was observed between the risk of invasive breast cancer and a history of GDM.	African American parous mothers with a history of GDM did not have an increased risk of breast cancer.
12	Brasky et al., 2013 [18]	USA	Case-control study (n = 2812)	The presence of hypertension, pre-eclampsia, gestational diabetes, or greater weight gain during pregnancy did not display any association with the risk of breast cancer.	Breast cancer risk was not linked to hypertension, pre-eclampsia, gestational diabetes, or increased weight gain during pregnancy
13	Perrin et al., 2008 [19]	Israel	Prospective cohort study (n = 37,926)	The incidence of breast cancer was higher in mothers who had GDM during pregnancy than those who did not. The cancer, however, did not develop before 50 years of age.	In postmenopausal mothers, GDM may be an early identifier of breast cancer.
14	Powe et al., 2017 [20]	USA	Prospective cohort study (n = 86,972)	5.96% of mothers reported a history of GDM. After 22 years of follow-up, 0.11% developed breast cancer with a history of GDM, while 2.62% developed breast cancer without a history of GDM. The history of GDM was associated with a reduced risk of developing invasive breast cancer.	Mothers with a history of GDM were not associated with an increased risk of developing invasive breast cancer
15	Wartko et al., 2017 [21]	USA	Population-based case-control study (n = 6336)	A history of GDM in mothers was found to be correlated with both endometrial cancer and endometrial hyperplasia. This association was not significant when adjusting for pre-pregnancy body mass index except for mothers under 50.	There was an association between endometrial cancer and endometrial hyperplasia and a history of GDM, particularly among younger mothers.
16	Liu et al., 2021 [22]	New Zealand	Case-control study (n = 650)	A history of GDM or large gestational delivery of an infant had a significantly increased risk of developing endometrial cancer up to two-fold.	Mothers who developed GDM during pregnancy had an increased risk of developing endometrial cancer.
17	Sun et al., 2021 [23]	China	Population-based cohort study (n = 15,941)	2.2% of patients had GDM, and 11% developed gynecologic cancers. There were no connections between GDM and the risks of any gynecologic cancers. GDM was linked to a higher risk of ovarian cancer in mothers with gestational hypertension.	There were no connections between GDM and the risks of any gynecologic cancers. The presence of gestational hypertension influenced GDM's impact on ovarian cancer risk.
18	Perrin et al., 2007 [24]	Israel	Prospective cohort study (n = 37,926)	In mothers who had been diagnosed with insulin-dependent GDM, none of them went on to develop pancreatic cancer, however, those with GDM did.	GDM is a risk factor for pancreatic cancer.
19	Marcoux et al., 2022 [25]	Canada	Retrospective cohort study (n = 1,030,537)	The incidence of cancer in the offspring exposed to GDM was 26.5 per 100,000 person-years. GDM was associated with the risk of any cancer, solid cancer, and blood cancer by 1.47, 1.44, and 1.61 times.	Hyperglycemia leads to an increased risk of childhood cancer and may be due to excess glucose being carcinogenic in utero.
20	O’Neill et al., 2015 [26]	UK	Case-control study (n = 127,248)	For certain types of cancer, an increase in birth weight was associated with an increased risk of developing cancer in the offspring.	There is a positive relationship between birthweight and several childhood cancers.
21	Seppala et al., 2019 [27]	Finland	Case-control study (n = 12,132)	GDM is linked to an elevated risk of childhood cancer in the offspring. The use of medications to treat GDM decreases the risk of childhood cancer. The incidence of leukemia was greater in the offspring exposed to a form of GDM.	Maternal diabetes is linked to an elevated risk of childhood cancer in the offspring.
22	Petridou et al., 2015 [28]	Sweden	Retrospective cohort study (n = 3,444,136)	The only associations that remained a risk factor for developing lymphoma were male sex and high birth weight.	GDM did not play a role in the development of childhood lymphoma.
23	Huang et al., 2022 [29]	USA	Retrospective cohort study (n = 2,245,941)	GDM in Denmark and Taiwan was not associated with cancer in offspring. However, the risk of glioma was slightly elevated in the offspring whose mothers had GDM.	There is no association, but there is some risk in the development of cancer in offspring.
24	Kessous et al., 2019 [30]	Canada	Retrospective cohort study (n = 236,893)	Malignancy diagnoses that lead to hospitalizations were compared for offspring born from mothers with GDM compared to those with healthy mothers.	GDM during pregnancy and childhood oncological hospitalizations were not associated.
25	Heck et al., 2015 [31]	USA	Case-control study (n = 426)	Underweight moms prior to pregnancy were related to the development of bilateral retinoblastoma. A positive trend of unilateral retinoblastoma was noted for moms with GDM.	Health factors of moms, such as being underweight and developing GDM, can lead to the development of retinoblastoma.
26	Podvin et al., 2006 [32]	USA	Population-based case-control study (n = 6545)	A significant positive relationship was found between the development of childhood leukemia and several factors, including advanced maternal age, birthweight 4 kg or more, neonatal jaundice, and Down syndrome. Some factors were protective against developing childhood leukemia, such as the mother being unwed and being of African American descent.	Advanced maternal age, neonatal birthweight 4kg or more, neonatal jaundice, and Down syndrome were associated with an increased risk of developing childhood leukemia.
27	Soegaard et al., 2018 [33]	Denmark	Retrospective cohort study (n = 1,187,482)	An offspring born to mothers with pregestational or GDM had a higher risk of developing childhood ALL. There was no association between childhood ALL and the later development of diabetes in mothers.	The overall risk of childhood ALL among the offspring of mothers with diabetes is low; certain aspects of the diabetic womb environment may encourage the development of ALL.

Discussion

Incidence and Type of Cancer in Mothers Who Develop GDM

Several studies have evaluated the incidence and prevalence of cancer following pregnancy in females who developed GDM intrapartum. Studies focus on either the development of cancer in mothers or their offspring. Before going into findings, it is imperative to remember that mothers who develop GDM typically tend to be older during their first birth, are obese, or are multiparous [7-9]. Several studies have found that GDM in mothers is associated with an increase in the risk of developing cancer [10-12]. The average incidence of overall cancer development was found to be 6.17%, ranging from 2.24% to up to 16% of sample populations [11-15]. One study observed the cumulative five-year incidence of cancer postpartum to be 7.48 per 1000 mothers in mothers who developed GDM [16]. Also, they observed that mothers with GDM who developed T2DM postpartum were 1.22 times more likely to develop any cancer [16]. Two studies, however, found there was no association between GDM and the development of postpartum cancer, with Bertrand et al. observing that of the patients who had developed cancer, only 4.9% had developed GDM during pregnancy [7,16].

One of the most evaluated cancers associated with GDM is breast cancer. Studies found that risk factors such as age of menarche, breastfeeding history, oral contraceptives, hypertension, weight gain, and pre-eclampsia did not vary between groups, nor did they present as risk factors for developing breast cancer [17,18]. Most studies found a significantly increased association with the risk of developing breast cancer, with an average of all studies having an incidence of 7.88% [9-13,15,19,20]. Park et al. observed that a singleton pregnancy with a GDM diagnosis was not associated with breast cancer; however, multiple gestations with GDM were associated with an increased risk of overall breast cancer. They noted a significant association between estrogen receptor-positive (ER+) and invasive breast cancer [15]. At the same time, there was only a slight association with developing ER-negative (ER-) breast cancer [15]. Bertrand et al. only noticed a significant increase in the risk of ER- cancer when birth was within 10 years of diagnosis [17]. However, Bertrand et al. and Powe et al. found no association between an increased risk of developing breast cancer, ER status, or invasive breast cancer in mothers with a history of GDM [17, 20]. Powe et al. observed that there may be a significant association between the risk of developing invasive breast cancer and mothers diagnosed with GDM who breastfed for less than six months during their lifetime [20].

Two studies, alternatively, found that there was up to a 14% decrease in the risk of developing breast cancer in mothers who developed GDM compared to mothers who did not [16,20]. Bejaimal et al. observed this to be true despite the fact that the mothers who developed GDM were more likely to have recently immigrated and lived in a lower-income neighborhood. At the same time, these mothers were likely to have primary care providers and more frequently visit their physicians before delivery, which could have significantly influenced successful monitoring [16]. Bertrand et al. went further and evaluated parous mothers with GDM compared to nulliparous mothers and observed that those parous mothers with GDM had a 34% and 18% decrease in the risk of in situ breast cancer and invasive ER+ breast cancer, respectively. They also found that parous mothers with GDM were 1.9 times more likely to develop invasive ER- breast cancer compared to nulliparous women [7].

The risk of gynecological cancers has also been evaluated, and one risk factor found in developing uterine cancer in mothers diagnosed with GDM was obesity [21]. Most studies found a significantly positive association between the risk of developing and the incidence of uterine cancers in mothers with GDM [10,21,22]. There is estimated to be over a two-fold increase in the risk of developing uterine cancer, with one study stating that 17.5% of mothers with endometrial cancer had GDM [22]. Gestational diabetes mellitus was also associated with a higher risk and incidence of ovarian cancer, with the presence of gestational hypertension furthering this risk [10,22,23]. However, Han et al. observed that there was no significant increase in the risk of developing ovarian cancer [11]. Concerning gynecological cancers as a whole, however, Sun et al. reported that there were no connections between GDM and any gynecological cancer [23]. In fact, they reported that the cumulative incidence of cervical cancer and uterine cancer was slightly lower in mothers with a history of GDM compared to those without [23].

Other cancers and their association with GDM, such as thyroid, have been analyzed. Several studies found there was a significant increase in the risk of developing thyroid cancer in mothers who developed GDM [11,12,14,16]. One study reported an increase of 24% in risk and found that mothers with GDM who went on to develop T2DM postpartum were 1.42 times more likely to develop thyroid cancer [16]. There was also a significantly increased risk of developing cancer in the digestive organs, specifically the pancreas [9,24]. The risk of nasopharyngeal cancer was also increased in one study; however, in Taiwan, there’s an increased incidence of Epstein-Barr virus, and further sub-analysis was not done [12].

Incidence and Type of Cancer in the Offspring of Mothers Diagnosed With GDM

Regarding the offspring of mothers with GDM, most studies focused on specific cancer types. However, one study focused on cancer incidence in general and observed that offspring from mothers who developed GDM have an incidence of 26.5 per 100,000 person-years of cancer [25]. Deleskog et al. found that the prevalence of cancer was higher in males at 55% than in females at 45%, with only one study agreeing that females have a higher prevalence [8,26]. A couple of studies reported that there was a strong association in the risk of developing childhood cancer, with Marcoux et al. stating an increased risk by 1.4 times [8,25,27]. This, however, was not agreed upon by Petridou et al., who found no association with cancer risk [28]. Marcoux et al., however, did not find that the strength of the association between childhood cancer and mothers who developed GDM decreased over time [25]. Huang et al. evaluated varying demographics and found that specifically in Denmark and Taiwan, a history of GDM in the mother was not associated with cancer in the offspring [29]. It is possible, according to one study, that the use of metformin to manage and treat GDM may be associated with a decreased risk of the development of cancer in the offspring [27].

When it comes to the development of cancer in the offspring of mothers who developed GDM, two studies observed no association with the risk, as the cumulative incidence of offspring cancer did not differ between mothers who developed GDM and healthy control mothers [28,30]. A couple of risks have been associated with the increased risk of cancer in the offspring of mothers who had GDM, such as offspring having an LGA, older mothers, and mothers who smoked [26,29]. O’Neill et al. observed that most cancer types were more prevalent in male offspring [26]. A couple of studies reported an increase in the risk of developing solid tumors, such as brain tumors like retinoblastomas and gliomas, and renal tumors such as nephroblastoma, up to 1.44 times the risk under the age of six [25,27,29,31]. An increase in birth weight was significantly associated with the risk of developing central nervous system (CNS) tumors and renal tumors [26]. For renal tumors, specifically, they were more prominent in female offspring [26]. However, Deleskog et al. observed no association with the risk of lymphoma and a decreased risk of up to 41% in CNS tumors in offspring whose mothers had GDM [8].

While Kesseous et al. observed no significant association with cancer risk, they did observe a slight increase in the risk of the offspring developing leukemia [30]. Several studies focused on leukemia, specifically ALL, and found that there was a significant increase in the risk of leukemia in the offspring of mothers with GDM, up to 47% [8,25-27,32,33]. Risk factors associated with ALL were offspring LGA, neonatal jaundice, trisomy 21, mothers having two or more abortions, and a high birth rate [8,25-27,32,33]. Concerning ALL specifically, there was also an increased risk of cancer development, with one study reporting an incidence of 2.8% [8,25,27,32,33]. Risk factors include advanced maternal age, a high birth rate, and LGA [27,32]. Huang et al., however, did not find a significant increase in ALL risk compared to mothers without GDM [29].

This review presents some limitations. One limitation is that the diversity in demographics of the selected articles is relatively limited when considering the demographics of the general population. In reading these publications, there was little to no mention of the baseline risk in the current demographic or demographic breakdown, which substantially contributes to the results of developing cancer. Better highlighting of the risk of GDM in these demographics would provide a stronger base to work from in determining the actual cancer risks. A suitable strength, however, is the study population in most articles, as they are typically grabbed from a network or database, which essentially covers their regional demographics. Future research should be done to determine risk conclusively and, if present, predictors that can be highlighted early on to prevent cancer development.

## Conclusions

Gestational diabetes mellitus is a critical condition that develops during pregnancy and is thought to be due to an increase in hPL, decreasing insulin sensitivity, and increasing insulin resistance. Maternal complications, such as an increase in the risk of developing T2DM, can occur, and neonatal complications arise from hypoglycemia, hypocalcemia, and LGA. The stress of pregnancy enhances GDM and has been shown to affect some mothers long-term postpartum permanently; studies have even shown a possible increase in the risk and incidence of cancer. The majority of studies included here observed an increase in the risk of developing breast cancer. There was also an increase in the risk of gynecological cancers, according to most studies, specifically uterine and ovarian cancer. Cancers not associated with the reproductive system include thyroid cancer, for which all studies evaluating it found an increased risk. A couple of studies evaluated the risk of cancer in the offspring of mothers with GDM, and most studies confirmed an association, with the most common cancer being ALL. Risk factors associated with the development of cancer in the setting of an offspring from a mother with GDM were increased maternal age, mothers who smoked, and LGA.

The publications used in the study lacked the aspect of confirming whether or not postpartum permanent sequels following a pregnancy with GDM occurred, especially in those who were diagnosed with cancer. While GDM is essentially a temporary condition that resolves following pregnancy in most patients, the possibility of contributing to future conditions years after its occurrence creates a sense of urgency and necessity to reduce the incidence of GDM. A glimpse of the limited studies done shows that there are some factors identified that contribute to the development of cancer when paired with GDM. Cancer is one of the leading causes of death. Further research to identify the risk factors or predictors associated with the development of cancer following GDM would enable reducing the prevalence of cancer in this patient population.
