# Correction: The Relationship Between Gestational Diabetes and the Risk of Cancer: A Systematic Review

**DOI:** 10.7759/cureus.c335

**Published:** 2025-10-01

**Authors:** Ethan Slouha, Kaitlyn M Gates, Hanin Al-Geizi, Esther Baah, Lucy A Clunes, Theofanis F Kollias

**Affiliations:** 1 Anatomical Sciences, St. George's University School of Medicine, St. George's, GRD; 2 Pharmacology, St. George's University School of Medicine, St. George's, GRD; 3 Microbiology, Immunology, and Pharmacology, St. George's University School of Medicine, St. George's, GRD

This article has been corrected to fix the following typo in the paragraph immediately preceding Figure 1: "...leaving 313 publications for full-text evaluation" corrected to "...leaving 340 publications for full-text evaluation."

